# Patatin‐domain‐containing (phospho)lipases under control: Mammalian co‐regulators and pathogenic activation mechanisms

**DOI:** 10.1002/2211-5463.70201

**Published:** 2026-01-31

**Authors:** Noopur Dubey, Lina Riegler‐Berket, Monika Oberer

**Affiliations:** ^1^ Institute of Molecular Biosciences University of Graz Graz Austria; ^2^ Field of Excellence BioHealth Graz Austria; ^3^ BioTechMed‐Graz Graz Austria

**Keywords:** (phosopho)lipase activation, ExoU, PNPLA2, PNPLA9, structure, VipD

## Abstract

Patatin‐like phospholipase (PNPLA) domain‐containing proteins are essential enzymes involved in lipid metabolism, membrane remodeling, and signaling pathways across various organisms. This review focuses on the structural and functional characteristics of four selected PNPLA proteins from different organisms with experimental 3D structures or biochemical data on protein–protein interactions that facilitate their co‐activation mechanism, namely VipD, ExoU, PNPLA9, and PNPLA2 (also known as ATGL). VipD and ExoU, phospholipases from *Legionella pneumophila* and *Pseudomonas aeruginosa*, respectively, are multidomain proteins and utilize distinct mechanisms for host cell interaction and pathogenesis. VipD binds to Rab proteins, underscoring the critical role of Rab5 in upregulating its enzymatic activity and contributing to the pathogenicity. ExoU requires ubiquitin for activation and exhibits an inhibited structure when complexed with its chaperone SpcU. PNPLA9, a calcium‐independent phospholipase A2, is predominantly expressed in the human brain, with mutations linked to neurodegenerative disorders and inflammation. The crystal structure of the Chinese hamster ortholog of PNPLA9 reveals a dimerization mechanism required for its catalytic activity, along with specific regions identified for membrane interaction and substrate binding. PNPLA2 is known for its triacylglycerol hydrolytic activity and is regulated by protein–protein interactions, particularly with the co‐activator ABHD5, which is crucial for its activation. This review highlights the diversity and conserved architectural segments of PNPLA proteins, reflecting their varied biological roles and regulatory mechanisms. Understanding the diverse protein–protein interactions that activate these enzymes is crucial for elucidating their roles in physiological and pathological contexts.

## Graphical abstract

Patatin domain‐containing (phospho)lipases are lipid‐hydrolyzing enzymes central to metabolism, membrane remodeling, and signaling. Their activity relies on precise co‐activation mechanisms involving protein–protein interactions and conformational rearrangements. Structural analysis reveals two key elements, named the D‐segment and the regulatory segment, that dynamically modulate active‐site architecture, control access to the substrate‐binding cavity, and enable formation of fully competent catalytic proteins.

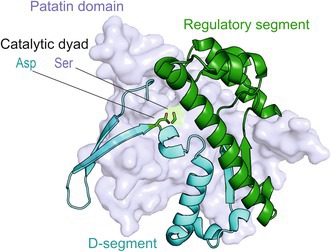

**Abbreviations**
3D: Three‐dimensional
ABHD5: Alpha/Beta Hydrolase Domain‐Containing Protein 5
AF: AlphaFold
ARs: Ankyrin repeats
ATGL: Adipose triglyceride lipase
ATP: Adenosine Triphosphate
CaM: Calmodulin
CG: *Cricetulus griseus*
CGI‐58: Comparative Gene Identification‐58
CTD: C‐terminal domain
DEER: Double electron–electron resonance
DNAJC5: DnaJ Heat Shock Protein Family Member C5
ExoU: Exoenzyme U
FABP4: Fatty Acid Binding Protein 4
G0S2: G0/G1 Switch Gene 2
HDX‐MS: Hydrogen‐Deuterium Exchange Mass Spectrometry
HILPDA: Hypoxia‐Inducible Lipid Droplet‐Associated Protein
iPLA₂β: calcium‐independent phospholipase A2 beta
LD: Lipid droplet
MLD: Membrane localization domain
NTD: N‐terminal domain
Pat17: Patatin 17
PI(4,5)P2: Phosphatidylinositol 4,5‐bisphosphate
PI3P: phosphatidylinositol 3‐phosphate
PLA: Phospholipase A
PLA2: Phospholipase A2
pLDDT: Predicted Local Distance Difference Test
PLIN1: Perilipin 1
PNPLA: Patatin‐like phospholipase
PPI: Protein–protein interaction
PROSITE: Protein Sites and Patterns Database
Rab: Ras‐related proteins
RMSD: Root‐mean‐square deviation
SDSL‐EPR: Site‐directed spin labeling electron paramagnetic resonance
SpcU: Specific Chaperone U
T3SS: Type III secretion system
TAGs: Triacylglycerols
VipD: vacuolar protein sorting inhibitor protein D

The Patatin‐like phospholipase (PNPLA) domain‐containing proteins are found in all forms of life, utilizing their lipase, phospholipase, and transacylase activities and consequently have major roles in signaling pathways, host colonization, sepsis, lipid metabolism, and membrane remodeling [1, 2, 3, 4, 5, 6].

The name of the family derives from ‘patatin’, a storage tuber protein that was first mentioned in a study reported in 1980, and for which the experimental three‐dimensional (3D) structure of an isoenzyme termed Pat17 was determined first in 2003 from the source organism *Solanum cardiophyllum* at the resolution of 2.2 Å [4, 7].

It is important to note that not all PNPLA domain‐containing proteins exhibit predominant phospholipase (PLA) activity. In this review, we use the nomenclature as listed in the PROSITE database of protein domains and functional sites, specifically PROSITE entry PS51635 annotated as ‘Patatin‐like phospholipase (PNPLA) domain profile’ [8].

In humans, the classic Ca^2+^‐independent phospholipase A2 family, also referred to as patatin‐like domain‐containing phospholipase A (PNPLA) family, is characterized by its lack of dependence on Ca^2+^ for activity or translocation [2, 9]. This PNPLA family consists of nine different members with different roles that are also distinguished into the lipase type, acting primarily on neutral lipids, and the phospholipase A (PLA) type, acting primarily on phospholipids [10].

Lipases and phospholipases are important enzymes in lipid metabolism or infection processes, and their activation often involves complex mechanisms, including protein activators or additional regulatory domains within the PNLPA domain‐containing proteins [3, 10]. PNPLA domain‐containing proteins are frequently regulated by slow, long‐term transcriptional mechanisms, as well as by faster post‐translational modifications (*e.g*., phosphorylation); neither mode of regulation is addressed here. Importantly, this review focuses on how (phospho)lipases often fine‐tune their hydrolytic activity through intricate networks of protein–protein interactions (PPI). These interactions result in proper localization of the (phospho)lipase in proximity of the substrate, *for example*, perilipins for human PNPLAs, DNAJC5 for ExoU, and we refer to excellent reviews elsewhere [11, 12, 13, 14]. PPIs also cause conformational changes within the active site residues, thus enabling elegant on–off switches for activity [11, 15, 16].

In addition to upregulation of activity, PPIs can also lead to inactive (phospho)lipases, lead to a tunable sequestering of the (phospho)lipase at different intracellular sites, or eventually to a complete off‐switch when the (phospho)lipases are targeted to degradation pathways, for example, via ubiquitination [17].

This review centers on PNPLA domain‐containing proteins, with an emphasis on how their structural features regulate activity mediated by PPIs (Fig. 1). We therefore selected three enzymes including bacterial and mammalian representatives for which experimentally determined three‐dimensional (3D) structures and a strong biochemical foundation for their activation mechanisms are available: ExoU (*Pseudomonas aeruginosa*), VipD (*Legionella pneumophila*), and mammalian PNPLA9 (represented by the crystal structure of the Chinese hamster (*Cricetulus griseus*) ortholog). Additionally, we address human PNPLA2, which lacks an experimentally determined structure to date but has been the subject of significant advances in elucidating its co‐activation mechanisms. Human PNPLA9 and the pathogenic proteins ExoU and VipD are phospholipases, whereas human PNPLA2 is primarily known for its role in intracellular lipolysis, acting on neutral lipids (triacylglycerols, TAGs) stored in lipid droplets [5, 10, 18, 19, 20, 21].

**Fig. 1:**
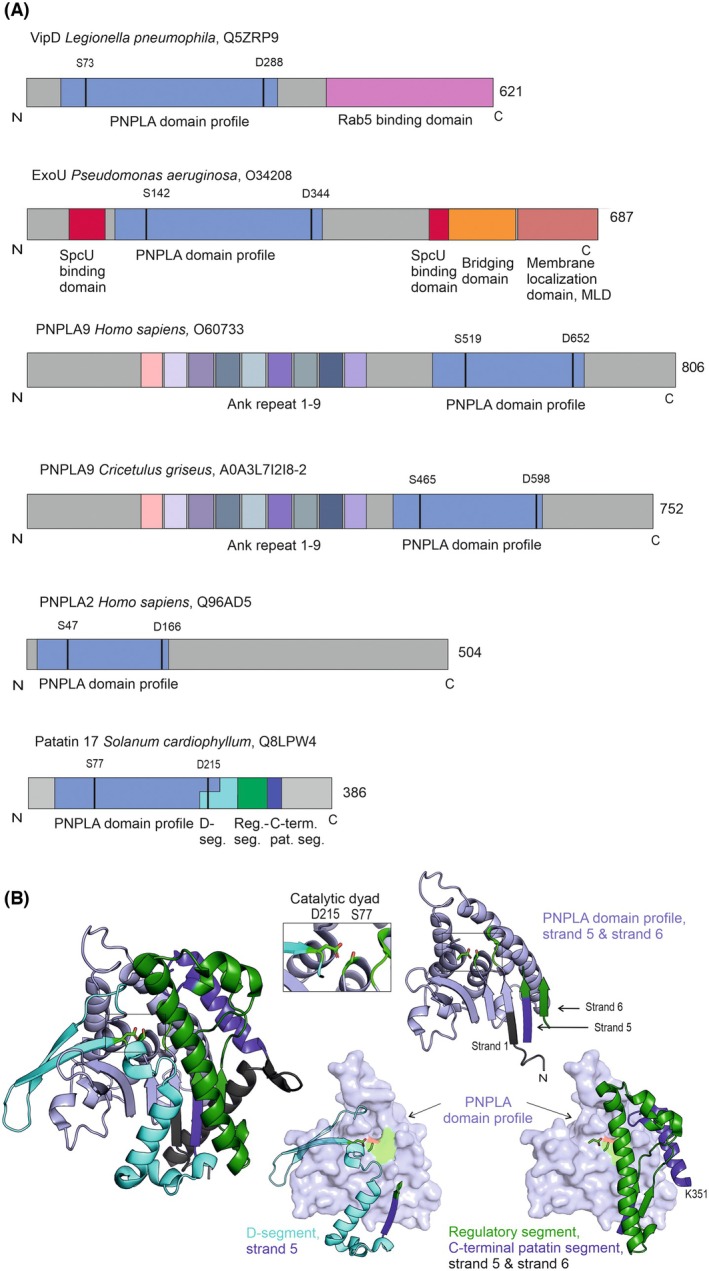
Domain architecture of various PNPLA domain‐containing proteins. (A) The PNPLA domain profile within the catalytic domain is shown in light blue, and the positions of the serine and aspartate residues forming the catalytic dyad are indicated. VipD from *Legionella pneumophila* features two main domains: the PNPLA domain profile region (light blue) and the Rab5‐binding domain (pink). ExoU from *Pseudomonas aeruginosa* is a multidomain protein comprising the PNPLA domain profile region (light blue), the SpcU‐binding domain (red), the bridging domain (orange), and the membrane localization domain (MLD, light brown), which collectively facilitate ExoU's phospholipase activity and membrane interaction. PNPLA9, from *Homo sapiens* and *Cricetulus griseus*, contain nine ankyrin repeats (depicted in different hues) in addition to the PNPLA domain profile region (light blue). PNPLA2 from *Homo sapiens* has the PNPLA domain profile region in light blue. Patatin 17 from *Solanum cardiophyllum* contains the PNPLA domain profile (light blue). Distinct segments important for comparing the co‐activation of the mentioned enzymes are indicated, including the D‐segment (cyan), the regulatory segment (green), and the structurally conserved C‐terminal patatin segment (blue). While these segments are present in VipD, ExoU, PNPLA9, and PNPLA2, they are omitted from the schematic representation there for readability. (B) A cartoon representation of Patatin 17 is shown, illustrating the three‐dimensional organization of the PNPLA domain profile (light blue), the D‐segment (cyan), the regulatory segment (green) and the C‐terminal patatin segment (blue). Separate images highlight the individual structural components. The catalytic dyad residues are depicted as green sticks.

Human PNPLA9 is primarily associated with lipid signaling and membrane phospholipid remodeling, important in maintaining neuronal health [9]. The phospholipases ExoU and VipD are bacterial virulence factors contributing to pathogenesis via different mechanisms, either by directly disrupting host membranes, as in the case of ExoU, or disrupting endosomal fusion and thus protecting the bacteria from degradation, as is the case for VipD [11, 22, 23].

The selected (phospho)lipases highlighted in this review share a PNPLA‐domain profile (PROSITE entry PS51635) and the ability to be activated and/or inhibited via PPIs, either directly by interaction with the PNPLA‐domain or other proteins. VipD and ExoU require host proteins for co‐activation to avoid self‐toxicity within the bacterial cells. Directly interacting and activating proteins for VipD are the Rab5 and Rab22 GTPases specific to eukaryotic endosomes. It is speculated that binding to the Rab‐proteins not only recruits VipD to the endosomes but also activates the phospholipase activity [22, 24]. ExoU requires interaction with ubiquitin as activator protein and phosphatidylinositol 4,5‐bisphosphate (PI(4,5)P2) as cofactor for full activation [25, 26, 27]. PNPLA9 is assumed to require dimerization for protein‐mediated activation [28]. ABHD5 acts as a co‐activator for PNPLA2 and thereby increases PNPLA2's basal TAG‐hydrolyzing activity [29, 30, 31, 32, 33]. In contrast to VipD and ExoU, both the enzyme and the co‐activator are expressed in the same cell and the same organism. PPI also leads to inhibition of PNPLA‐domain‐containing (phospho)lipases: The chaperone SpcU from the pathogen is a directly interacting and inhibitory protein for ExoU—an intriguing mechanism that also protects *P. aeruginosa* from the toxic activity of ExoU [34, 35]. No direct inhibitory protein for VipD is reported. G0S2 and HILPDA are prominently reported as direct protein inhibitors of human PNPLA2, whereas calmodulin binding inhibits the activity of PNPLA9 [22, 24, 28, 31, 33, 36, 37]. We review similarities and differences in the co‐activation mechanism often inferred from structural, biochemical, and *in silico* studies and combine this information to propose a mechanism for co‐activation of PNPLA2, which currently lacks experimental 3D structures. We also discuss the name‐giving protein patatin from plants as example of active PNPLA‐domain profile containing lipase, but it is important to note that no specific co‐activating protein is known to date.

## The PNPLA domain is essential for catalytic function and harbors important segments for regulating the hydrolytic activity

The PNPLA domain is the critical site of a finely tuned response to physiological stimuli mediated by PPI. Patatin harbors a fold distinct from the α/β hydrolase fold with a Ser‐His‐Asp/Glu catalytic triad and is related to that of human cytosolic phospholipase A2 (cPLA2) [4, 38, 39].

Patatin has approximately three layers, αβα with a central, mostly parallel β‐sheet (Fig. 1B). In some PNPLA‐domain‐containing proteins, the sandwiching α‐helices are more pronounced on one side of the central sheet. The hydrolytic reaction is carried out in an interplay of three conserved motifs, namely (i) an evolutionarily conserved glycine‐rich region that is also involved in forming the oxyanion‐hole, (ii) a serine lipase consensus motif GXSXG containing the nucleophile, and (iii) a conserved DGA/G‐motif. The side chain of the aspartic acid (proton acceptor) in the DGA/G motif is aiding in the deprotonation of the catalytic serine required to initiate the reaction (see Fig. 1 in [12]).

In the first half‐reaction, the nucleophilic serine attacks the carbonyl carbon of the lipid substrate ester bond, forming an acyl‐enzyme intermediate complex, which is stabilized by the oxyanion hole. The resulting alcohol‐containing fragment (glycerol‐ester or lysophospholipid) is released. In the second half‐reaction, termed deacylation step, a water molecule serves as the nucleophile, attacking the acyl‐enzyme intermediate to release a free fatty acid and regenerate the enzyme (recently reviewed in [12]).

Our understanding of the underlying mechanisms of activation and inhibition varies between these different lipases and is based on outstanding studies, including a vast array of biochemistry and integrated structural biology methods by dedicated research groups worldwide. Experimental 3D structures have been determined only for a handful of PNPLA‐domain‐containing lipases including the name giving protein Patatin‐17 from *Solanum cardiophyllum* (PDB IDs: 1OXW [4], 4PK9, 4PKA, 4PKB [38]); ExoU from *Pseudomonas aeruginosa* (3TU3 [35], 4AKX [40]); ExoU from *Pseudomonas fluorescens* A506 (4QMK [41]; VipD from *Legionella pneumophila* (4KYI [24], 4AKF [22]); the Chinese hamster ortholog of PNPLA9 termed calcium‐independent phospholipase A2 beta from *Cricetulus griseus* (CGsh‐PNPLA9, PDB code: 6AUN, short form, UniProt: A0A3L7I2I8–2, [28]) as well as PlpD from *Pseudomonas aeruginosa* (5FQU, 5FYA [42]). No experimental 3D structure of human PNPLA2 or any orthologous proteins is available.

**Fig. 2:**
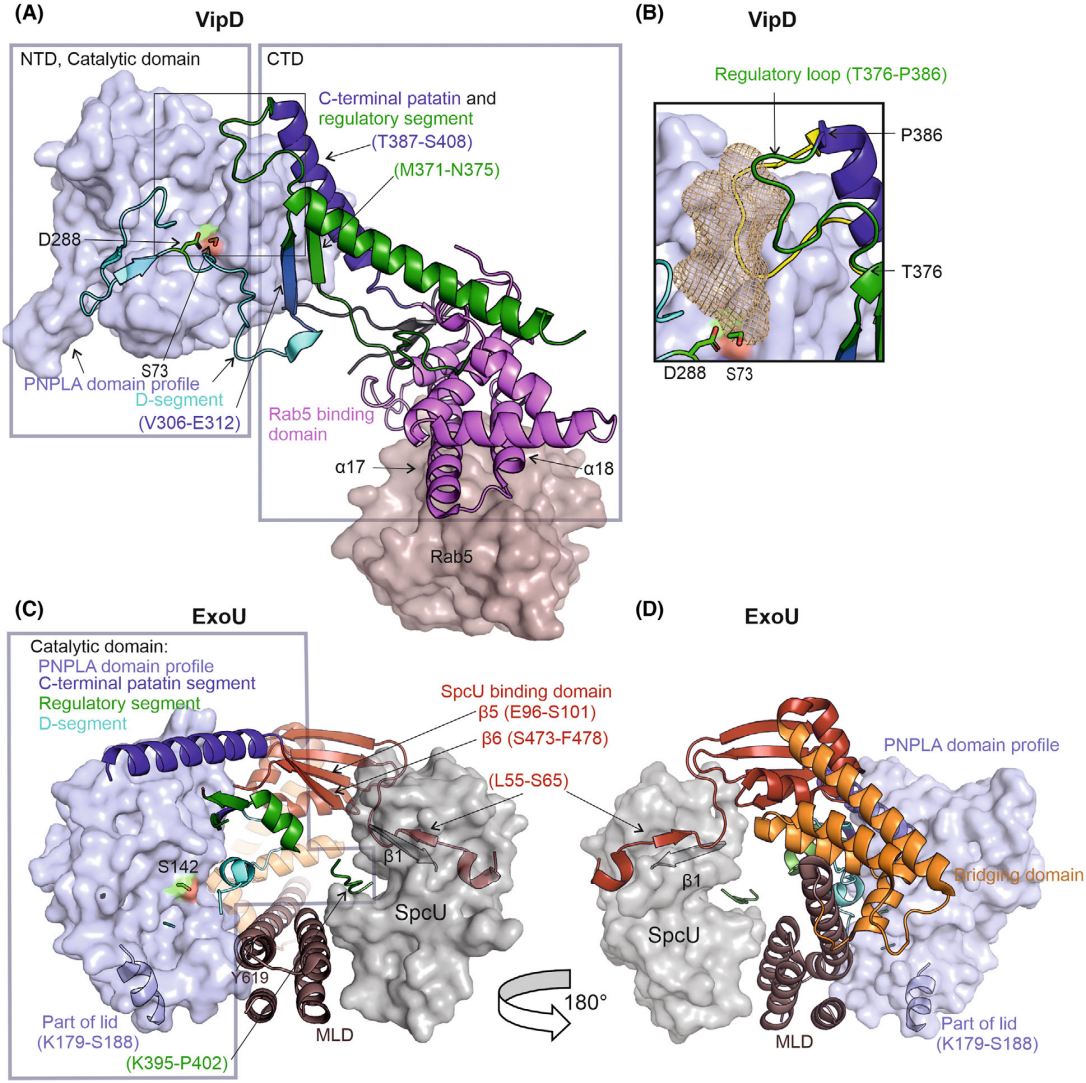
Structural insights into VipD and ExoU. (A) The active form of VipD in complex with Rab5 (PDB: 4KYI) is shown, highlighting its domain architecture. The N‐terminal catalytic domain contains the PNPLA domain profile (light blue surface) and the D‐segment, which is shown as cyan cartoon. The active site residues, Ser73 and Asp288, are depicted as green sticks. The so‐called C‐terminal domain includes the regulatory segment (green) and the C‐terminal patatin segment (blue). Additionally, key strands (Met371‐Asn375) and helices (Thr387‐Ser408) that strongly interact and complement the N‐terminal domain are highlighted. The Rab5‐binding domain is shown as a pink cartoon, with two helices (α17, α18) specifically interacting with Rab5, which is depicted in brown (surface representation). (B) Conformational changes in VipD upon Rab5 binding are demonstrated by the superposition of the active (green/blue, bound, 4KYI) and inactive (yellow, unbound, 4AKF) conformations of VipD. The loop spanning residues Thr376‐Pro386 transitions to an open conformation, facilitating substrate entry. The cavity is depicted as brown mesh. (C) The ExoU structure in complex with SpcU (PDB: 3TU3) reveals its domain organization. The catalytic domain contains the PNPLA domain profile, displayed as a light blue surface, while additional structural elements are depicted as cartoons. These include the D‐segment (cyan; note that electron density for the surface loop containing the active Asp598 is missing), the regulatory segment (green), and the C‐terminal patatin segment (blue). Other structural domains are shown as follows: the SpcU‐binding domain (red), the bridging domain (orange), and the membrane localization domain (MLD, brown). SpcU is depicted in gray (surface representation), with its β1 strand interacting with the Leu55‐Ser65 region of the SpcU‐binding domain of ExoU. Furthermore, another interaction of SpcU with the loop Lys395‐Pro402 from the regulatory segment of ExoU is highlighted. Tyr619 in the membrane localization domain (MLD), also involved in ubiquitin binding, is indicated. (D) A 180° rotated view of ExoU in complex with SpcU (PDB: 3TU3) is shown. The domains and segments are depicted using the same color scheme as in panel C.

In the PNPLA‐domain‐containing proteins discussed herein, four strands of the central 6‐stranded β‐sheet are formed by the PNPLA domain profile (ProSITE entry PS51635). The latter has a length of approx. 180 residues, is placed in different regions of the proteins (Fig. 1, Table 1), and harbors a cavity enclosed by five positionally conserved helices, the catalytic nucleophile, the oxyanion‐hole, and is lined by helices, loops, and parts of the central strands. Strands 5 and 6 are not part of the PNPLA domain profile. To improve clarity and facilitate comparisons, we introduce the concept of a ‘D‐segment’, ‘Regulatory segment’, and a ‘C‐terminal patatin segment’ in this review. The D‐segment is defined as an amino acid stretch that brings the catalytic Asp in spatial proximity to the catalytic Ser and then leads to Strand 5 in the central sheet adjacent to Strand 1. It overlaps partly with the PNPLA domain profile, yet the lengths of the D‐segment vary in PNPLA‐domain‐containing proteins, leading to apparently longer PNPLA domain profiles. In Pat17, the N‐terminal part of the D‐segment (D‐segN) harbors an additional two‐stranded antiparallel β‐sheet (Fig. 1B). The C‐terminal part of the D‐seg (D‐segC) of Pat17 forms two helices on the D‐segment side of the protein, whereas the structures of ExoU and VipD (see below and Fig. 2) reveal predominantly unstructured regions within D‐segC on this side of the central sheet [4, 22, 24, 40]. Sequentially and spatially adjacent to Strand 5 is the ‘regulatory segment’. It is rather diverse with respect to sequence and structure in PNPLA‐domain‐containing proteins and emerges from Strand 5. The regulatory segment then leads via different trajectories to the C‐terminal patatin segment, which forms one of the sandwiching helices and is present in all proteins discussed here (Fig. 1B). In Pat17 and VipD, long helices are present in the regulatory segment [4, 22, 24, 38]. In VipD, this helix plays a critical role in co‐activation. In PNPLA9, a long helix of the regulatory segment and a helix of D‐segC are involved in dimerization and membrane binding, both critical for phospholipase activity [28]. In ExoU, large parts of this regulatory segment are highly flexible. Parts of the regulatory segment further complement the central β‐sheet with a parallel Strand 6 adjacent to Strand 5 in the experimental structures of Pat17, PNPLA9, VipD, and ExoU. The regulatory segment can be modeled only with lower confidence scores in human PNPLA2 and does not form an explicit strand 6.

**Table 1:** PNPLA‐domain‐containing proteins and the positions of the different segments as well as accession codes in the PDB and UniProt.

Protein	PNPLA‐domain profile (PS51635)	D‐segment	Regulatory segment	C‐term. Patatin segment	PDB‐code (resolution)	UniProt ID
Pat17	32–229	191–255	257–329	330–352	4PK9 (1.96 Å)	Q8LPW4
VipD	38–301	270–304	311–382	383–411	4KYI (3.08 Å) 4AKF (2.90 Å)	Q5ZRP9
ExoU	107–357	319–370	375–445	446–471	3TU3 (1.92 Å) 4AKX (2.94 Å)	O34208
iPLA2β/CGsh‐PNPLA9	427–611	585–632	638–720	721–739	6AUN (3.95 Å)	A0A3L7I2I8–2
PNPLA2	10–179	149–180	183–230	231–254	–	Q96AD5

## VipD

Upon inhalation of *Legionella pneumophila*, the bacteria are primarily phagocytosed by alveolar macrophages, which also serve as the primary site of replication of the pathogen in the lungs. Once inside, *L. pneumophila* employs sophisticated strategies to avoid destruction and create a replicative niche within the host cells. The vacuolar protein sorting inhibitor protein D (VipD, UniProt ID: Q5ZRP9) harbors a PNPLA domain and is involved in subverting host cell defense [43, 44]. VipD is injected into the cytoplasm of the host cell through the Type IV secretion system [24]. Inside the cell, VipD binds to GTP‐bound forms of endosomal Rab5 and Rab22 proteins of the host, thereby activating the phospholipase activity of VipD for hydrolysis of phosphatidylinositol 3‐phosphate (PI3P), a key lipid in endosome function. This mandatory activation mechanism depending on host proteins is crucial in preventing self‐damage to *Legionella*. Additionally, the binding of VipD to Rab5 or Rab22 inhibits the interaction of Rab proteins with their downstream effector molecules, which are important for endosomal trafficking. Consequently, VipD‐mediated changes in the lipid and protein composition of endosomal membranes prevent the fusion of *Legionella*‐containing vacuoles and host endosomes, thereby protecting the bacteria from degradation [22, 24].

VipD (621 amino acids) is encoded on the open reading frame lpg2831 of the *Legionella pneumophila* genome. VipD specifically targets the sn‐1 position of phospholipids (*e.g*., from PI3P), typical lipids on early endosomes, classifying it as a PLA1 phospholipase [24, 45]. A short N‐terminal amphipathic α‐helix (termed N18, covering residues Met1‐Asn16) is suggested to facilitate the positioning of the phospholipase domain toward the membrane, since deletion of this helix leads to loss of PLA1 activity [22, 24].

VipD consists of two structural, interconnected domains, namely an N‐terminal domain (Met1‐Ala316, NTD) and a C‐terminal Rab5 binding domain (Phe317‐His621, CTD) (Fig. 2A,B; 4AKF [22], 4KYI [24]). The PNPLA domain profile of VipD is in the NTD, located between residues Leu38 and Leu301, with active site residues Ser73 and Asp288, and the glycine‐rich motif Gly42‐Gly47 that also forms the oxyanion hole (Fig. 1). A long stretch from Ala270‐Glu304, including the D‐segment, positions Asp288 close to the catalytic Ser73. D‐segC (Asp288‐Glu304) is unstructured and traverses on the side of the PNPLA domain to the central β‐sheet without forming α‐helices that would sandwich the central β‐sheet (Fig. 2A). VipD‐CTD contains mostly α‐helices, very few β‐strands, and unstructured regions at the very C terminus (46 C‐terminal residues were even deleted for crystallization, PDB‐ID: 4AKF, resolution 2.9 Å [22]). VipD‐CTD is crucial for the tight interaction with Rab5 and Rab22 proteins [22].

This structural organization, including the interaction between these two domains, is significant for VipD's biological activity and Rab5‐mediated mechanism of activation in human host cells. The two domains interact strongly via interactions within the central β‐sheet and α‐helices (Fig. 2A). Of high relevance for regulation is the regulatory segment that finishes the patatin‐domain by complementing the central β‐sheet of VipD‐NTD (Val306‐Glu312) as Strand 6; even so formally it is assigned as β‐strand (Met371‐Asn375) of VipD‐CTD (Fig. 2A). Furthermore, an α‐helix (Thr387‐Ser408) of VipD‐CTD forms the C‐terminal patatin segment, thus interacting with the N‐terminal domain (Fig. 2A). Of special relevance is a loop region (Thr376‐Pro386). In this review, we call it ‘regulatory loop’, as it is a portion of the regulatory segment, yet it is also called ‘lid’ in the literature. In the apo‐form of VipD (PDB‐ID: 4AKF [22]), this regulatory loop is located in close proximity to parts of the D‐segN, leading to Asp288 (Ala270‐Ile290) from VipD‐NTD, and consequently closes off the active site (Fig. 2A,B). Since the remaining cavity is small (443 Å^3^) and inaccessible for phospholipid substrate, this form very likely represents the inactive conformation. The crystal structure (PDB ID: 4KYI, resolution 3.08 Å) of VipD (aa19‐564) in complex with Rab5 (216 aa, UniProt ID P51148) reveals a beautiful ‘heterotropic allosteric activation mechanism’ [24]: Upon Rab5 binding, the regulatory loop undergoes conformational rearrangement and is repositioned, thus exposing a large substrate‐binding pocket within the catalytic domain. This cavity is easily accessible from the surface and is large enough (735 Å^3^) to bind an acyl‐chain of the substrate and directly connects to the active site residues. Very likely, this open conformation represents the catalytically active conformation of VipD (Fig. 2B, [24]). These drastic conformational changes in the regulatory loop result from cumulative movements of a series of very small conformational changes of the CTD and structural elements in the regulatory segment connecting the NTD and CTD of VipD.

Apart from the regulatory loop, the structure of VipD in the VipD‐Rab5 complex compared to the free VipD protein is essentially unchanged [22, 24]. The Rab5 interaction surface of VipD is a helical hairpin located in the CTD of VipD at the most distal end, far from the active site in the NTD (Fig. 2A). The binding interface in VipD involves residues in helices called α17 (Phe442, Ala446, Ala450, and Leu454) and helix α18 (Tyr473, Ile480, and Val483), with the distance from the catalytic Ser73 to Tyr473 being larger than 50 Å. In human Rab5, a conserved aromatic patch (F58, W75, and Y90) is essential for interaction with VipD, the same region that would also be critical for the binding of its downstream effectors [22, 24].

## ExoU

Exoenzyme U (ExoU) is encoded within the *Pseudomonas aeruginosa* ExoU operon on the main circular genome along with the protein ‘specific Pseudomonas chaperone for ExoU’ (SpcU, GenBank U97065.1). A type 3 secretion system (T3SS) delivers the phospholipase ExoU into the cytoplasm. Upon injection into the mammalian host, ExoU localizes to the plasma membrane by interacting with PI(4,5)P2, a highly abundant lipid in the plasma membrane of eukaryotic cells [46]. Then, ExoU acts as a PLA2‐like toxin by cleaving host membrane phospholipids, for example, phosphatidylcholine at the sn‐2 position, thereby disrupting the plasma membrane, interfering with cellular signaling, and promoting cell necrosis [13, 34].

Crystal structures of ExoU (UniProt ID: O34208; 687 aa) in complex with SpcU (UniProt ID O66100, 137 amino acids) revealed a multidomain domain architecture (PDB ID: 3TU3, resolution of 1.9 Å [35], 4AKX, heterodimeric complex, 2.94 Å [40]). A noncomplexed form is only available for ExoU from *P. fluorescens* A506 (4QMK, 45.3% sequence identity, 2.5 Å [41]). ExoU has four major domains listed below, namely the SpcU‐binding domain, the catalytic, the bridging, and the membrane localization domain (Figs 1, 2C,D).

The very N terminus of ExoU is largely disordered, with the first 65 N‐terminal residues of ExoU containing polar amino acids essential for recognition by the T3SS [13]. Following this region is the SpcU‐binding domain, which is divided into two segments formed by residues Pro56‐Arg101 and Ala472‐Glu502. The complex structure of ExoU bound to SpcU (4AKX) shows close interaction between the two proteins. Residues Leu55‐Ser65 of ExoU participate in β‐strand complementation with the β1‐sheet of SpcU (Fig. 2C,D, [35]). Additionally, strand complementation occurs between the two segments of the SpcU‐binding domain. Specifically, Glu96‐Ser101 (β5) interact with residues Ser473‐Phe478 (β6). This strand complementation is observed in both the free and SpcU‐complexed structure.

The catalytic domain contains the PNPLA domain profile (PS51635) of ExoU, which spans residues Leu107‐Ile357. Due to high flexibility, many loops are not visible in the ExoU crystal structures. In the ExoU‐SpcU complex structures 4AKX and 3TU3, these include Glu189‐Lys201, which could be located on top of the catalytic site [35, 40]. Due to the flexibility and positive charges within this region, it can be speculated that the region (Lys179‐Lys201) forms a lid that might aid in substrate recruitment of the phospholipase, or is involved in forming open and closed conformations in a similar manner as observed in gastric lipase and lysosomal lipase [47, 48, 49].

The active site Ser142 is in spatial proximity to the glycine‐rich region (Gly111‐Gly116) that also forms the oxyanion hole. The D‐segment is highly flexible; electron density cannot be traced in D‐segN (Ser329‐Gly345, including the catalytic Asp344) in the ExoU (4QMK, [41]) or ExoU‐SpcU complex structures (4AKX, 3TU3 [35, 40]). D‐segC is a long loop region that adopts only one helical turn before re‐entering the central sheet at Asp370 where strand 5 is added to the central sheet (Fig. 2C). The patatin structure is then completed by the regulatory segment (that also forms strand 6) and by the C‐terminal patatin segment, which is dominated by a long helix on the sandwiched side of the central β‐sheet.

The bridging domain (Leu481‐Met580) is crucial for binding the activator ubiquitin [50]. Currently, there is no experimental structure of ExoU in its active state, namely in complex with its co‐activator. Both linear and lysine‐linked polyubiquitin chains are involved in various cellular processes that may regulate this activity. Kinetic studies demonstrated that the affinity for ubiquitin increases with chain length, with Lys48‐linked tetra‐ubiquitin showing higher affinity than linear tetra‐ubiquitin, and octa‐ubiquitin exhibiting the highest overall affinity [25]. Mutations in the hydrophobic patch of ubiquitin (Leu8, Ile44, and Val70) negatively impact ExoU binding and/or activation [25].

The membrane localization domain (MLD, Ser587‐Thr687) adopts a four‐helix bundle structure and interacts with the negatively charged PI(4,5)P2 at the inner leaflet of the host cell [35, 40]. Additionally, MLD is also reported to be important for interaction with ubiquitin, *for example*, with residues Arg661 and Tyr619 (see Fig. 2C) [25]. Zhang et al. indicated that Arg661, located within a loop that lacks electron density, also contributes to ExoU oligomerization, highlighting another unsolved question in the field [51].

Structural insights into the binding of ExoU with its co‐activators, ubiquitin and PI(4,5)P2 resulting in conformational changes are gradually emerging. It was proposed that ExoU can associate with either the membrane or ubiquitin independently. While ubiquitin binds to the bridging domain and the MLD, it does not significantly affect ExoU's activity in the absence of PI(4,5)P2 [52]. The prevailing model suggests that ExoU first binds to ubiquitin in the cytoplasm at the bridging domain. Following this activation, ExoU associates with PI(4,5)P2 via the MLD, leading to the localization of the catalytic domain at the plasma membrane [13, 34, 52, 53, 54].

Approximately 25% of the ExoU structure is disordered, including several key functional segments. A nonmodeled 30‐residue stretch in the D‐segment (D‐segN; Gly321‐Met348), which includes the catalytic Asp344, remains unresolved due to insufficient electron density. Additional disordered regions include large portions of the regulatory segment (Gly378‐Leu394, Ala402‐Leu412, and Pro426‐Met446; corresponding residues Pro374‐Phe392 in ExoU from *Pseudomonas fluorescens* where it is traceable except for five residues) and a 13‐residue basic‐hydrophobic loop within the MLD (Ala660‐Thr672, Fig. 2C,D). The latter is essential for PI(4,5)P2 binding and is on the same face of the protein as the catalytic Ser142 and Asp344. This region becomes conformationally restricted upon binding of ExoU to both liposomes and diubiquitin [52, 53].

Interestingly, a short peptide is sandwiched between ExoU and SpcU in the complex structures, yet the identity is debated since the peptide is attributed either to the N‐terminal region (Gly28‐Gln33) or parts of the disordered regulatory segment (Lys395‐Pro402), which is also not resolved in the uncomplexed structure [35, 40, 41]. According to an AlphaFold model of ExoU, this region belongs to the missing ExoU region Lys395‐Pro402 as already interpreted in this manner earlier by Halavaty *et al*. [35].

Insightful site‐directed spin labeling electron paramagnetic resonance (SDSL‐EPR) and double electron–electron resonance (DEER) studies suggest that parts of the regulatory segment of ExoU (Leu427‐Met446, no electron density) bind both the membrane and ubiquitin, undergo conformational rearrangement that forms a substrate‐binding pocket and thereby enable full hydrolytic activity and membrane disruption [27, 53, 54, 93].

ExoU undergoes oligomerization upon binding to PI(4,5)P2, further stabilizing the active conformation yet remains poorly understood despite increasing biophysical and computational analyses. While PI(4,5)P2 alone can induce oligomerization, full enzymatic activity requires both cofactors acting synergistically [51, 55].

Such examples of activation by oligomerization exist in both bacterial and eukaryotic phospholipases [53, 56, 57]. Although it offers valuable clues, the oligomerization of ExoU upon binding to PI(4,5)P2 at the cell membrane remains poorly understood.

## PNPLA9

PNPLA9 belongs to a subgroup of group VI calcium‐independent PLA2s and is also referred to as calcium‐independent phospholipase A2, PLA2G6 or iPLAβ [58, 59]. Members of this group do not require Ca^2+^ for activity or membrane binding and differ from calcium‐dependent phospholipase A₂s in structure and catalytic residues [58, 59]. The intracellular lipase is encoded on chromosome 22 (22q13.1 with 16 exons) and detected at very high levels in the human brain, yet also in different human tissues [60]. PNPLA9 (Uniprot: O60733, (Human Protein Atlas accession: ENSG00000184381‐PLA2G6* [61]) harbors the PNPLA domain profile in the C‐terminal half (Leu481‐Met665) and hydrolyzes membrane phospholipids, lysophospholipids, and cardiolipins, thus generating signaling lipids critical for stress response [2].

In humans, mutations in the corresponding gene are associated with neurodegenerative disorders, inflammation, as well as imbalances in membrane remodeling and calcium homeostasis [62]. Dimerization, membrane association, covalent modification close to the active site by acyl‐CoA, relief from inhibition by calmodulin, and interaction with ATP are reported to increase the activity of PNPLA9 [28].

**Fig. 3:**
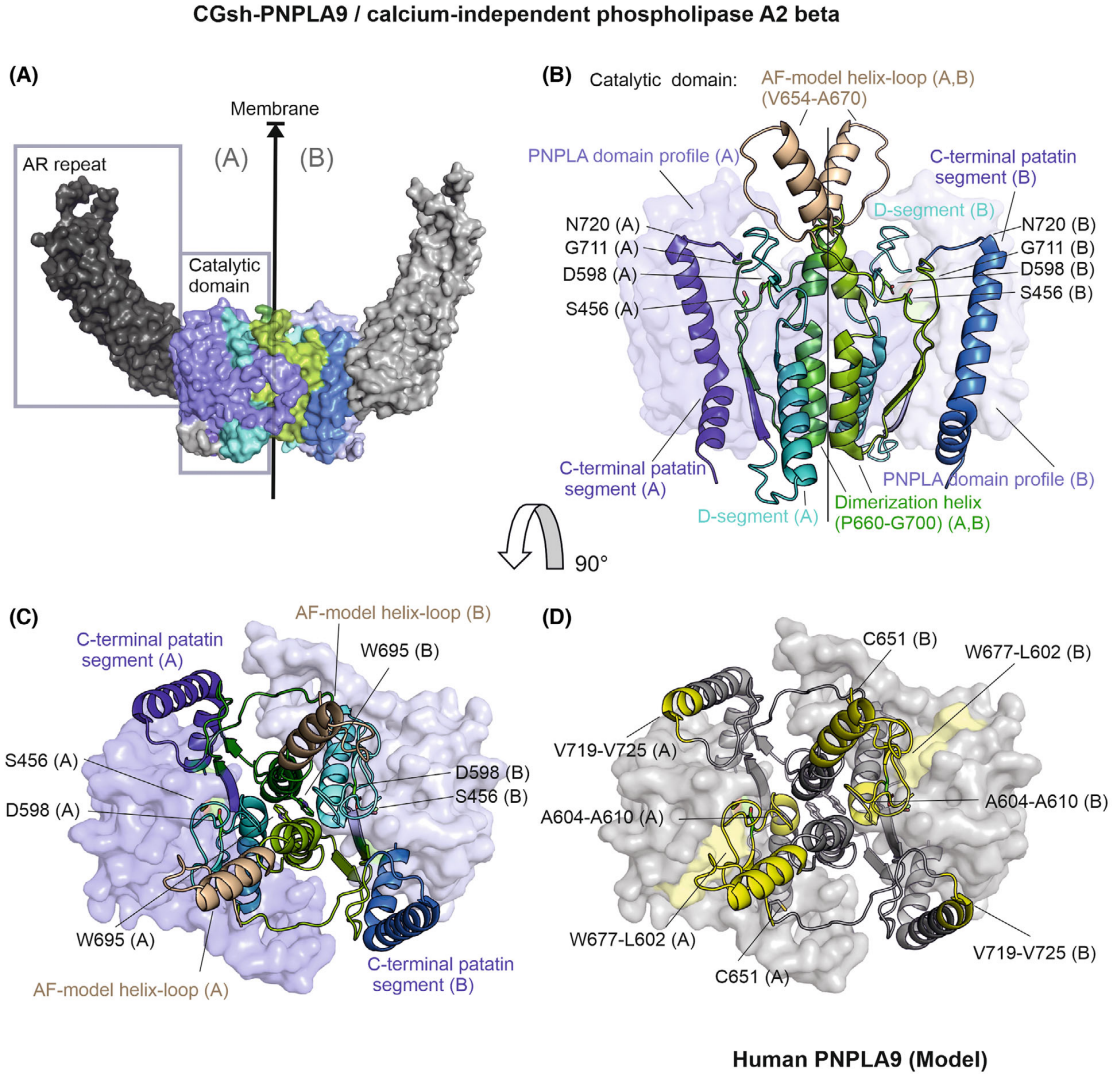
PNPLA9 forms a homodimer in its active form. (A) The surface representation of the short form of PNPLA9 from *Cricetulus griseus* (CG‐shPNPLA9, PDB: 9AUN) illustrates the catalytic domain with distinct color coding: light blue for the PNPLA domain profile, cyan for the D‐segment, green for the regulatory segment, and blue for the C‐terminal patatin segment. Ankyrin repeats are depicted in gray. This color scheme is consistently applied across panels A–C. The dimerization plane is indicated by an arrow, which also reflects the orientation toward the membrane. (B) A close‐up view of the catalytic domain (same orientation as panel A) depicts the active site residues located on the membrane‐facing surface. The PNPLA domain profile is shown as a transparent surface. The D‐segment, the C‐terminal patatin segment, the dimerization helix in the regulatory segment, and the helix–loop motif (the position of residues Val654‐Ala670 is taken from the AlphaFold model) are presented as cartoon. Notably, the helix–loop motif lacks observable electron density. Active site residues are represented as green sticks. Chain B is distinguished by lighter hues compared to chain A. (C) The catalytic domain, rotated 90° relative to the view in panels A and B, displays the catalytic center as it appears from the membrane's perspective. Residues Trp695 of both chains are highlighted; when these are exchanged to glutamate, homodimerization and enzymatic activity are abolished. (D) In the same orientation as panel C, residues corresponding to human PNPLA9, that were identified as protected upon membrane binding in HDX experiments, are marked in yellow. Additionally, residue Cys651 is depicted as a stick representation. Cys651 is also located on the membrane‐facing side of the protein and has been reported to activate PNPLA9 upon autoacylation.

Although experimental structures are lacking for all nine human PNPLA proteins, valuable insights can be inferred from the X‐ray structure of a shortened variant from the Chinese hamster (*Cricetulus griseus*) ortholog for PNPLA9 (PDB‐Code 6AUN, UniProt A0A3L7I2I8–2, termed SH‐iPLA2β, CGsh‐PNPLA9, Fig. 3). This isoform has 91% sequence identity to the short isoform of human PNPLA9 and contains 752 instead of 806 residues, with Gln396 replacing a 54‐amino‐acid insert. The numbering used herein corresponds to the residues observed in the crystal structure of CGsh‐PNPLA9 (3.95 Å, 6AUN [28]).

The structure of CGsh‐PNPLA9 reveals an unstructured N terminus, 9 helical ankyrin repeat (AR) regions, a linker, and the catalytic domain at residues Arg421‐Pro752 (Figs 1A, 3). Residues Met1‐Ala80 and several loop regions, including region Val652‐His670, are unmodelled in the crystal structure, indicative of flexible regions [28]. The PNPLA domain profile (PS51635) region is within residues Leu427‐Met611 with a glycine‐rich sequence (Gly431, Gly432, Gly433) as well as catalytic dyad forming residues Ser465 and Asp598. The active site pocket is fairly accessible to the surface at the membrane‐exposed side [28]. Localization to the membrane is essential for the catalytic function of PNPLA9. In human PNPLA9, the most prominent membrane interaction regions are residues Val708‐Met730, predicted to adopt a helix–loop region as studied by HDX‐MS [2, 63, 64]. In CGsh‐PNPLA9, these residues correspond to Val654‐Ala670, corresponding to a region not seen in the crystal structure due to disorder resulting in lack of electron density. For this review we modeled the helix–loop region using AlphaFold3 and included it in our figures (Fig. 3B–D). Further protection in human PNPLA9 upon membrane binding can be seen for residues Trp631‐Leu655 (corresponding to CGsh‐PNPLA9: Trp577‐Leu602), Asn658‐Ala664 (CGsh‐PNPLA9:Asn604‐Ala610), and Val773‐Leu778 (CGsh‐PNPLA9: Val719‐Val725), all in loop regions close to the active site on the surface of CGsh‐PNPLA9, bringing the substrate and active site in spatial proximity (Fig. 3D).

CGsh‐PNPLA9 appears to require dimerization for full activity, which might be a possible explanation why no extra activation domain or activator protein is observed. Dimer formation is suggested to enable cooperative activation, transacylation, and acyl transfer reactions. The crystal structure reveals dimerization via the catalytic domains, including a long helix involved in the dimer interface (Fig. 3B–D). Gly640 is at the beginning of a loop region, followed by a long α‐helix (Pro660‐Gly700) essential for dimer formation that is interrupted by a short kink region (Trp681‐Gly686) [28]. Parts of this helix correspond to the membrane‐binding region (CGsh‐PNPLA9: Val654‐Ala670) identified by HDX‐MS on human PNPLA9 [58, 64].

The variant Trp695Glu renders CGsh‐PNPLA9 mostly monomeric and lacks phospholipase activity [28]. Residues Gly532‐Gln574 form an extensive, stacked loop region with large parts reaching over to the other monomer. The two active site residues (Ser465, Asp598) are relatively close (distance Ser465 (chain A) – Ser465 (chain B): 30 Å, Fig. 3B). Asp598 is within the D‐segment (Ala585‐Asn605) that continues into a sandwiching α‐helix (Thr607‐Lys624) forming part of the dimer interface. Previous reports hypothesized ankyrin‐mediated oligomerization; however, this hypothesis is not supported by the experimental structure that shows the ankyrin repeats peripherally positioned to the dimer interface formed by the catalytic domain [28].

Additionally, other post‐translational activating modifications of CGsh‐PNPLA9 are reported: Autoacylation of a thiol (Cys651, Fig. 3D) in the presence of acyl‐CoA has been shown to activate the phospholipase [65]. Cys651 resides in the unresolved membrane‐binding region, approximately 20 Å from the active site. The covalent attachment of a long fatty acid chain via autoacylation of Cys651 is proposed to enhance membrane binding and/or induce conformational changes. This activation is suggested to occur even in the presence of the inhibitory protein calmodulin (CaM), potentially resulting in increased phospholipid turnover [28]. Interaction of PNPLA9 with CaM in the presence of Ca^2+^ inhibits the activity of CGsh‐PNPLA9. The binding site for calmodulin is still controversial and is suggested to reside in the catalytic domain or the N‐terminal ARs [28, 64, 65]. Very recent cross‐linking and co‐immunoprecipitation studies indicated that the PNPLA2 co‐activator ABHD5 interacts with human PNPLA9, yet these findings need further corroboration [66].

The N‐terminal ARs are suggested to play regulatory roles via different mechanisms. Based on the X‐ray structure, ATP binding is suggested in the AR region, whereas different loci had been suggested previously [28]. Furthermore, the ARs orient toward the membrane‐binding interface and might be functional in binding to different cellular membranes. Cleavage of the ARs increases activity, suggesting an autoregulatory mechanism of these helical repeats, *for example*, via restricting access to the membrane. This is reminiscent of the reported increased lipolytic activity of PNPLA2 upon cleavage of the helical and unstructured C‐terminal half of the protein [33, 58, 67]. The available crystal structure of CGsh‐PNPLA9 cannot resolve 81 residues at the very N terminus, which might play a crucial role for subcellular localization of CGsh‐PNPLA9 and therefore influence hydrolytic activity [28]. ARs are a versatile motif mediating PPI. It is unknown whether more PPIs lead to oligomerization of PNPLA9 on the membrane or whether the ARs have additional roles in the regulation of PNPLA9 activity.

## PNPLA2/ATGL

Human PNPLA2 is an intensively studied PNPLA family member also known as ATGL, desnutrin, FP17548, iPLA2zeta, or TTS‐2.2. It is encoded on chromosome 11p15.5, and various laboratories confirmed hydrolytic activities for triacylglycerols (TAGs), retinyl esters, phospholipids, as well as transacylase activities to generate or remodel fatty acid esters of hydroxy fatty acids [29, 68, 69, 70, 71]. While patients with mutations in the gene suffer from TAG accumulation in multiple tissues, selective inhibition of PNPLA2 has positive health effects regarding insulin resistance and liver inflammation. The activity of PNPLA2 is controlled at transcriptional, translational, and post‐translational levels [20, 29, 30]. Differential localizations of PNPLA2 and ABHD5 are key prior to the actual co‐activation by ABHD5: In the basal state, ABHD5 interacts with perilipin 1 (PLIN1), thus sequestering ABHD5 and preventing interaction with PNPLA2. Activation of β‐adrenergic receptors leads to phosphorylation of PLIN1, ABHD5, and the lipases PNPLA2 and HSL by protein kinases leading to dissociation of the ABHD5‐PLIN1 complex and enabling ABHD5 to engage and co‐activate PNPLA2 [1, 15, 19, 29, 72, 73, 74].

**Fig. 4:**
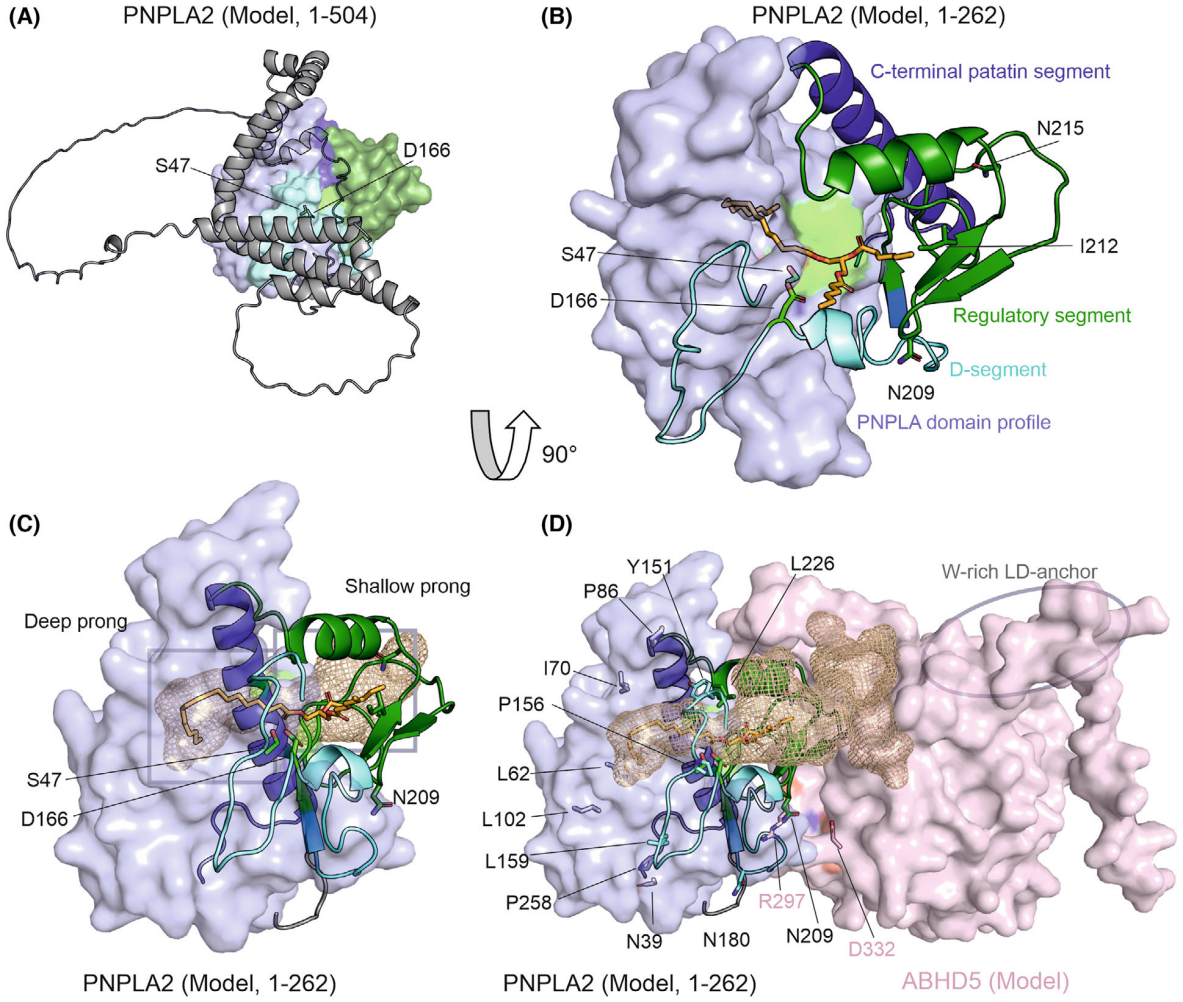
Predicted structures of human PNPLA2 and the PNPLA2‐ABHD5‐complex. For all panels, the color scheme is as follows: PNPLA domain profile, light blue surface; D‐segment, cyan cartoon; regulatory segment, dark green cartoon; C‐terminal patatin segment, blue cartoon; catalytic dyad, green sticks. (A) Full‐length PNPLA2 model. The patatin core—including the D‐segment, regulatory segment, and C‐terminal patatin segment—is shown in surface representation, while residues Leu254‐Leu504 are displayed as a gray cartoon. (B) PNPLA2 model (residues 1–262) in the same orientation as panel A. The substrate triacylglycerol (TAG) is shown in the binding pocket as orange sticks, and the catalytic residues Ser47 and Asp166 are depicted as green sticks. Residues in the regulatory segment implicated in ABHD5 interaction (Asn215, Ile212, Asn209) are highlighted. (C) PNPLA2 model (residues 1–262) rotated 90° relative to panels A and B. The binding pocket is shown as a brown mesh, and the deep and shallow prongs are indicated, with an inflection point around the catalytic residues (green sticks). (D) PNPLA2–ABHD5 complex model in the same orientation as panel C. The substrate‐binding cavity is shown as a brown mesh. Residues that affect activity or activatability (Ile70, Tyr151, Pro156, Leu159, Asn180, Gly189, Asn209, Ile212, Asn215, Leu226, Pro258) and residues with no detectable effect on ABHD5 binding (Asn39, Leu62, Leu102) are highlighted. Key ABHD5 residues required for PNPLA2 binding, Arg297 and Asp332, are shown as sticks, along with the tryptophan‐rich LD‐anchor at the ABHD5 C‐terminus.

Human PNPLA2 is a 504 amino acid protein (UniProt Q96AD5, ProteinAtlas: ENSG00000177666‐PNPLA2) with the PNPLA domain profile region within residues Ile10‐Lys179 (PS51635) (Figs 1, 4). No experimental 3D structure of human PNPLA2 is known; predicted AlphaFold (AF) models have very high and high confidence scores (pLDDT) for Arg4‐Asn252 and less confidence for the rest of the protein. The term 3D model refers to models we prepared specifically for this review and those published previously [33, 75, 76, 77]. The structure of the PNPLA domain profile residues has not changed from homology models to different AF versions. Differences in the models can be seen in the regulatory segment and especially in the localization of the C‐terminal half of the protein. Modeled residues C terminus of Leu254 harbor helical and long unstructured regions with lower confidence scores, for which the positional placements vary drastically between models (Fig. 4A). These regions are probably flexible without a binding partner (proteins, membranes, lipid droplets). It is interesting to note that the 3D models do not have predicted strand 6 in the central β‐sheet; even so, the regulatory segment (Thr183‐Glu239) is predicted to form a separate 2‐stranded sheet (Fig. 4B). Whether some of the predicted C‐terminal helices of PNPLA2 adopt similar positions and function as the ARs of CGsh‐PNPLA9 or whether they are reminiscent in function to perilipins is unknown. Surface accessibility of the C‐terminal half of PNPLA2 is corroborated by multiple phosphorylation sites, namely Thr370, Ser404, Ser407, Ser428, Ser476, and Thr490 (according to UniProt Q96AD5).

In the N‐terminal catalytic domain Met1‐Leu254, the 3D model shows a large, two‐pronged fork cavity with a deep prong formed by α‐helices and a shallow prong along the surface of the molecule forming a large semi‐open ligand binding groove (Fig. 4B,C) [75, 76]. The active site residues Ser47 and Asp166 are located in proximity to the inflection region between the deep and shallow cavity. Gly14‐Gly19 are part of the Gly‐rich region predicted to form the oxyanion‐hole. The AF3 model with a TAG as substrate was specifically calculated for this review and shows that the oleic acid at the sn‐2 position reaches into the deep prong, whereas the other two alkyl moieties adopt different positions in the shallow prong (Fig. 4B,C). In this arrangement, Ser47 is at a position enabling a nucleophilic attack on the ester bond of the substrate. PNPLA2 in basal conditions preferentially cleaves TAG at the sn‐2 position; however, cleavage at the sn‐1 position is also observed in the presence of the co‐activator ABHD5/CGI‐58 [78].

In *in vitro* experiments, residues Met1‐Leu254 are sufficient for PNPLA2's hydrolytic activity, co‐activation by the protein ABDH5/CGI‐58, and inhibition by the proteins G0S2 or HIG2/HILDPA [30, 33, 36, 37, 79, 80]. In early work, Schweiger *et al*. had shown an increase *in vitro* activity and increased binding to ABHD5/CGI‐58 upon truncation of 220 residues C terminus of PNPLA2 [67]. While the scaffolding helices and strands of the deep binding pocket for PNPLA2 are predicted with very high confidence within the N‐terminal PNPLA domain profile, Tyr176‐Leu255 of the regulatory segment has lower scores and also less conservation among different animals [76]. Previous homology modeling approaches yielded different placements of the regulatory segment and included more helical regions [21, 33, 72, 73, 74, 76, 77]. Very recently, we showed that the regulatory segment, especially residues Asn209‐Ala215 (Fig. 4B), is important for co‐activation by ABHD5; AF‐modeling of a triple mutation variant showed conformational rearrangements of region Asp197‐Leu216 forming a loop and a long α‐helix [76].

The α/β hydrolase domain‐containing protein 5 (ABHD5, UniProt Q8WTS1, 349 aa), also termed Comparative Gene Identification‐58 (CGI‐58), not only acts as a protein co‐activator for PNPLA2, but also for PNPLA1 and PNPLA3 [12, 81, 82, 83, 84]. ABHD5 has been shown in different studies to directly interact with PNPLA2 and thus increase lipolytic activities [19, 21, 29, 33, 72, 73, 77, 81]. The co‐activation of PNPLA2‐mediated TAG‐hydrolysis by ABHD5 is assumed to work best at an equimolar concentration of PNPLA2 [18, 30].

No experimental structure for ABHD5 is available, yet the predicted model shows that it contains a tryptophan‐rich N‐terminus acting as a LD‐anchor (Fig. 4D), the remainder is predicted to adopt an α/β hydrolase‐like fold with a cap‐domain [85, 86, 87]. ABHD5 does not have a catalytic nucleophile and accordingly lacks activities toward small‐molecule ester substrates or acylglycerols. Interestingly, Jebessa *et al*. reported a serine protease activity, whereas previously reported acyltransferase activity and lysophosphatidic acid acyltransferase are disputed and/or disproven [88].

The PPI surface of the PNPLA2‐ABHD5 complex is subject of ongoing research. Elegant studies revealed that residues corresponding to Arg297, Gly326, and Asp332 of human ABHD5 (some experiments were performed with the mouse ortholog) are spatially close and involved in PNPLA2 interactions [31, 89]. The regulatory segment of PNPLA2, including Asn209, Ile212, and Asn215, plays an important role in co‐activation, despite the observation that single or even triple amino acid exchanges in PNPLA2 did not impede the interaction with ABHD5 [76]. Modeling of the PNPLA2‐ABHD5 complex for this review suggests that the shallow prong of PNPLA2's substrate‐binding pocket is embraced by ABHD5 in a packman‐like fashion with an extensive PPI interface, highly similar to the predicted interface of PNPLA1 with ABHD5 (Fig. 4D, [76, 83, 90]). Asn209, Ile212, and Asn215 of PNPLA2 and Arg297, Gly326, and Asp332 of ABHD5 are part of the large interaction surface in agreement with these experiments [31, 32, 76]. Exchange of Tyr151 and Leu226 resulted in drastically reduced basal activity, yet that remaining activity could be nicely stimulated by ABHD5 [76]. Residues identified by mutational scanning of PNPLA2 revealed that variants Ile70Ala, Pro86Lys, and Leu159Ala had markedly reduced basal activity and could only be ‘moderately’ activated by ABHD5 [91]. Both sets of exchanges are located in loops and helices related to the deep prong of the binding pocket. Exchanges Asn39Glu, Leu62Lys, and Leu102Lys on the opposite side of substrate‐binding area did not significantly impact ABHD5 binding, which is also in good agreement with the predicted model [91]. Kohlmayr *et al*. also identified PNPLA2 variants with reduced binding of CGI‐58, namely: Pro156Lys, Leu159Ala, Asn180Leu, Gly189Glu, Pro258Leu, Leu261Glu, Lys275Ala and especially Phe348Glu and Arg351Leu. According to the PNPLA2 model, these residues are localized at different position of the surface of PNPLA2: Gln180 and Gly189 are both at the predicted PNPLA2‐ABHD5 interaction interface, whereas Pro156 and Leu159 are in the N‐terminal part of the D‐segment at a different side of the protein (Fig. 4D). Interestingly, lysates of PNPLA2 variants Phe348Glu + Arg351Leu showed good basal activity, yet reduced co‐activation by ABHD5, along with microscopy data indicating loss of lipolytic activity [91]. The 3D model does not predict any interaction between ABHD5 and the flexible C‐terminal half of ATGL, yet any interpretation of the C‐terminal half (also including Pro258, Leu261 and Lys265) is still highly speculative at this stage.

While a high‐resolution experimental structure of the PNPLA2–ABHD5 complex would provide the most definitive insights, ongoing biochemical and structural studies continue to refine our understanding of PNPLA2 activation. Based on current data, we suggest that activation may involve conformational or allosteric changes within PNPLA2, enhanced substrate delivery, and/or facilitated product release mediated by the co‐activator ABHD5. Notably, ABHD5 lacks intrinsic catalytic activity but likely harbors a lipid‐binding cavity, consistent with a role in lipid trafficking rather than hydrolysis [76]. Enhanced product release would be expected to alleviate product inhibition, a model supported by the increased hydrolytic activity observed in the presence of FABP4 and by experimentally demonstrated interactions between ABHD5 and FABP4 [85]. Structural models of the PNPLA2–ABHD5 complex further indicate a substantial expansion of the lipid‐binding cavity, potentially representing a shared substrate or product exchange interface (Fig. 4D and [76]). In addition, the physiological role of the C‐terminal half of PNPLA2 remains insufficiently defined and warrants further investigation. Finally, dimerization of PNPLA2—analogous to that observed for PNPLA9—may represent an additional regulatory mechanism contributing to enzyme activation, although this possibility remains speculative and awaits experimental validation.

## Conclusions

**Fig. 5:**
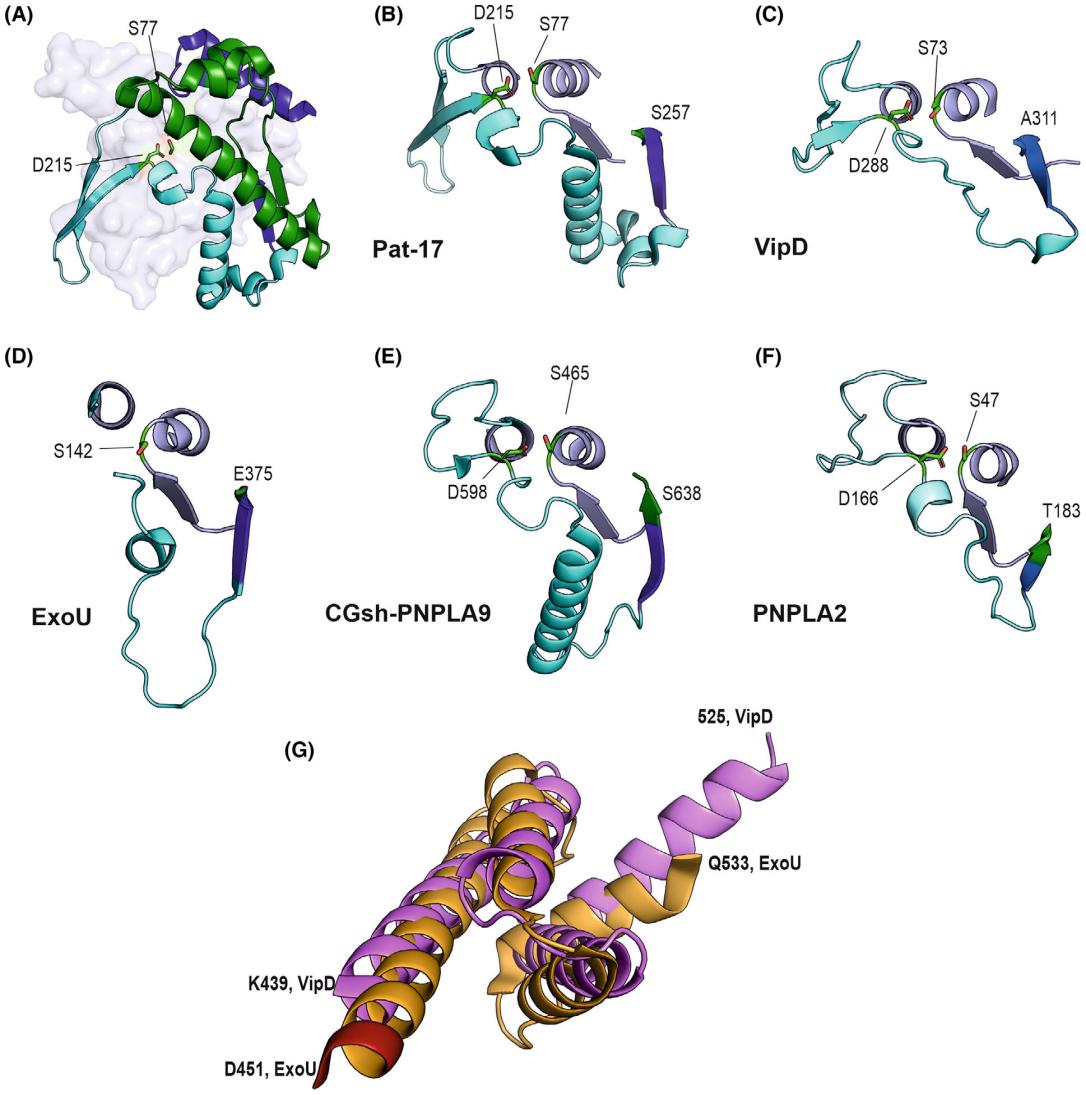
Similarities and differences in co‐activation of PNPLA‐domain‐containing proteins. (A) Pat17 D‐segment (cyan), regulatory segment (green) and C‐terminal patatin segment (blue) of Pat17 in cartoon representation. The PNPLA domain profile shown as transparent cartoon; (B–F) Cartoon of the D‐segment (cyan) to the start of the regulatory segment (green) in addition to residues forming the nucleophilic elbow. The catalytic dyad residues Ser and Asp are depicted as green sticks, when they could be modeled in the X‐ray structures. B: Pat17 (4PK9); C: VipD (4KYI); D: ExoU (3TU3), E: CGsh‐PNPLA9 (6AUN); F: PNPLA2 (AF‐model). (G) Similarity of helical scaffolds used for binding of ubiquitin in ExoU (bridging domain, orange, Gly454‐Gln533) and the Rab5 binding domain of VipD (pink, Lys439‐Ala525).

Many PNPLA‐domain‐containing proteins require interaction with (co)‐activating proteins to achieve high hydrolytic activity. No protein activator is known for Patatin from *Solanum cardiophyllum*, which therefore can be seen as the prototype of an intrinsically catalytically active PNPLA protein. Comparison of Pat17 with VipD, ExoU, CGsh‐PNPLA9/iPLA2β, and the predicted structure of PNPLA2 reveals different segments (PNPLA domain profile, D‐segment, regulatory segment (after strand 5), and the C‐terminal patatin segment) with distinct features (Fig. 1). Residues of the PNPLA domain profile build 4 strands of the central sheet, 5 helices that form a large part of the substrate‐binding pocket and harbor the catalytic Ser and Asp residues and the oxyanion‐hole. The general architecture of the PNPLA core region is conserved in all analyzed proteins. Strand 5 and the C‐terminal patatin segment are also structurally highly conserved. These are in stark contrast to the D‐segment that includes the catalytic Asp (and is overlapping with the PNPLA‐domain profile at this region) and the regulatory segments, which are characterized by high flexibility, conformational changes, or completely disordered regions in the absence of the respective activator proteins (Figs 1, 2C and Fig. 5). In this brief summary, we want to highlight and compare similarities and differences in the co‐activation mechanisms described in detail above, which have evolved to effective activation strategies in the distinct physiological niches.

- Positioning of the catalytic residues: The catalytic serine and the oxyanion hole are structurally constrained by the conserved architecture of the PNPLA domain profile, although the catalytic aspartate located within the D‐segment displays pronounced conformational flexibility (Fig. 5A–F). PPI or dimerization is therefore suggested to stabilize and rigidify the catalytic aspartate in a conformation competent for hydrolysis. In CGsh‐PNPLA9, the N‐terminal portion of the D‐segment (D‐segN) is positioned directly at the dimerization interface and lacks defined secondary structure elements. Consequently, D‐segN likely requires stabilization via PPI or dimerization. In line with this notion, an amino‐acid exchange within the regulatory segment that is also positioned at the dimer interface results in predominantly monomeric and catalytically inactive CGsh‐PNPLA9 [28]. Both apo‐ExoU and the ExoU in complex with its inhibitor SpcU lack electron density for large portions of the D‐segment, including the catalytic aspartate, indicating extensive disorder and explaining the inactive state of the enzyme (Fig. 5D). It is therefore likely that binding to ubiquitin and to the membrane induces a conformational rearrangement that stabilizes the catalytic dyad thereby enabling catalysis. The 3D structure of activated ExoU is not available.
- Positioning of the regulatory segment, including change of access to the substrate‐binding site: The regulatory segment of VipD contains a flexible loop (Thr376‐Pro386) that undergoes pronounced conformational changes upon Rab5 binding, thereby permitting access to the substrate‐binding pocket, as demonstrated by experimentally determined crystal structures (Fig. 2B). In CGsh‐PNPLA9, the residues Gly711‐Asn720, which correspond to the flexible loop of VipD, adopt a conformation intermediate to the apo‐ and Rab5‐bound states observed for VipD (Fig. 3). Compared to Pat17, the active site of CGsh‐PNPLA9 is more accessible and readily accommodates phospholipids with long polyunsaturated fatty acid chains. In ExoU, large portions of the regulatory segments cannot be modeled in currently available X‐ray structures, which capture only the inactive states of the enzyme (Fig. 2 [35, 40]). In human PNPLA2, the regulatory segment includes residues that are essential for co‐activation, and amino acid substitutions in this region are also shown to reduce binding to ABHD5 (Fig. 4 [75, 76]). In the current model, access to the shallow prong of the PNPLA2‐ABHD5 substrate‐binding pocket is unobstructed and can accommodate bulky lipid substrates supporting both hydrolytic and transacylation reactions (Fig. 4). It remains unclear whether segments of the PNPLA2 regulatory segment complement the central β‐sheet by contributing to the canonical strand 6, and also whether the helix and adjacent loop within the regulatory segment modulate substrate access in a manner analogous to the experimentally observed mechanism in VipD.
- Positioning of the (phospho)lipase at the site of the substrate: Efficient catalysis requires that enzymes gain physical access to their substrates. The native substrates of VipD, ExoU, PNPLA9, and PNPLA2 are not water soluble but are embedded in cellular membranes or localized to specific organelles such as lipid droplets or membranes. Therefore, these (phospho)lipases must be appropriately positioned at these sites to exert their catalytic activities. Changes in subcellular localization are mediated by post‐translational modification, *for example*, phosphorylation events that are essential for PNPLA2, and are coupled to conformational rearrangements in distinct regions of the PNPLA‐containing proteins. VipD harbors an N‐terminal amphipathic helix implicated in membrane association. In CGsh‐PNPLA9, a membrane‐interacting loop region within the regulatory segment has been proposed to insert to the membrane based on biochemical and biophysical studies (Figs 2, 3); however, this region is not resolved in the available crystal structure. ExoU localizes to the membrane through interaction with the inner‐membrane component PI(4,5)P2 via a dedicated MLD‐domain (Figs 1, 2). Interestingly, PI(4,5)P2 also induces oligomerization of ExoU, which may not only enhance membrane association but also increase the conformational stability of otherwise flexible loop regions, such as the D‐segments. For PNPLA2, lipid droplet targeting is often associated with the enigmatic C‐terminal domain. Nevertheless, *in vitro* studies demonstrate both basal activity and ABHD5‐dependent co‐activation of truncated PNPLA2 variants lacking this region. Collectively, previous and current observations underscore the importance of the co‐activators ensuring proper localization and activation, with ABHD5 itself likely contributing localization determinants that are also essential for efficient PNPLA2 function [85, 86, 87, 89].
- Positioning of the co‐activator binding domains: The phospholipases ExoU and VipD both harbor dedicated co‐activator binding domains (Figs 1, 2) for their strictly host‐derived co‐activators. Both the ubiquitin binding region in the bridging domain of ExoU and the Rab5 binding domain of VipD are composed of similarly arranged α‐helical bundles, exhibiting a root‐mean‐square deviation (RMSD) of 3.8 Å over 64 aligned residues (Fig. 5G). This structural similarity suggests that these domains may represent related scaffolds for co‐activator recognition and binding [92]. In contrast, no specialized co‐activator binding domain has been identified for PNPLA9, and it remains unclear whether predicted helical elements in the C‐terminal half of PNPLA2 form a comparable scaffolding platform that facilitates interaction with its co‐activator ABHD5.

Continued experimental efforts by the scientific community will delineate the protein–protein interfaces, key residues, and structural domains that govern localization, co‐activation, and allosteric regulation across distinct PNPLA family members and co‐activation mechanisms.

## Funding

This research was funded by Austrian Science Fund (FWF), Grant SFB Lipid Hydrolysis DOI 10.55776/F73, doc.fund Biomolecular Structures and Interactions – BioMolStruct DOI 10.55776/DOC130 (both MO). LRB is the recipient of an APART‐MINT Fellowship of the Austrian Academy of Sciences at the Institute of Molecular Biosciences of the University of Graz.

## Conflict of interest

The authors declare no conflict of interest.

## Author contributions

ND, LRB, and MO analyzed the literature. MO calculated new 3D models. ND, LRB, and MO analyzed and interpreted the models. ND, LRB, and MO wrote the paper.
